# A comparison of statistical methods for modeling count data with an application to hospital length of stay

**DOI:** 10.1186/s12874-022-01685-8

**Published:** 2022-08-04

**Authors:** Gustavo A. Fernandez, Kristina P. Vatcheva

**Affiliations:** School of Mathematical and Statistical Sciences, University of Texas Rio Grande Valley, One West University Boulevard, Brownsville CampusBrownsville, TX 78520 USA

**Keywords:** Count data, Poisson regression, Negative binomial regression, Zero-inflated Poisson regression, Zero-inflated negative binomial regression, Simulation study

## Abstract

**Background:**

Hospital length of stay (LOS) is a key indicator of hospital care management efficiency, cost of care, and hospital planning. Hospital LOS is often used as a measure of a post-medical procedure outcome, as a guide to the benefit of a treatment of interest, or as an important risk factor for adverse events. Therefore, understanding hospital LOS variability is always an important healthcare focus. Hospital LOS data can be treated as count data, with discrete and non-negative values, typically right skewed, and often exhibiting excessive zeros. In this study, we compared the performance of the Poisson, negative binomial (NB), zero-inflated Poisson (ZIP), and zero-inflated negative binomial (ZINB) regression models using simulated and empirical data.

**Methods:**

Data were generated under different simulation scenarios with varying sample sizes, proportions of zeros, and levels of overdispersion. Analysis of hospital LOS was conducted using empirical data from the Medical Information Mart for Intensive Care database.

**Results:**

Results showed that Poisson and ZIP models performed poorly in overdispersed data. ZIP outperformed the rest of the regression models when the overdispersion is due to zero-inflation only. NB and ZINB regression models faced substantial convergence issues when incorrectly used to model equidispersed data. NB model provided the best fit in overdispersed data and outperformed the ZINB model in many simulation scenarios with combinations of zero-inflation and overdispersion, regardless of the sample size. In the empirical data analysis, we demonstrated that fitting incorrect models to overdispersed data leaded to incorrect regression coefficients estimates and overstated significance of some of the predictors.

**Conclusions:**

Based on this study, we recommend to the researchers that they consider the ZIP models for count data with zero-inflation only and NB models for overdispersed data or data with combinations of zero-inflation and overdispersion. If the researcher believes there are two different data generating mechanisms producing zeros, then the ZINB regression model may provide greater flexibility when modeling the zero-inflation and overdispersion.

## Background

In healthcare, length of stay (LOS) is a key indicator used to assess the hospital care management efficiency, cost of care, quality control, appropriate use of hospital services and resources, and hospital planning [1–6]. The need for efficient hospital management has been exemplified with the recent onset of the 2019 coronavirus/COVID-19 pandemic. Health crises like these show the best interest of patients, hospitals, and public health is in the efficient management of hospital stays while ensuring adequate bed capacity and that clinician time can be provided for patients with other conditions [7]. Reducing LOS improves financial, operational, and clinical outcomes by decreasing the costs of care for a patient and minimizing the risk of hospital-acquired conditions [8, 9]. In some hospitals, administrators benefit from using predictive models to assist with planning and resource allocation for deliveries [9]. Clinics optimize clinical settings by implementing analytical applications leading to timely and accurate decision making while reducing the hospital LOS [8–10]. Hospital LOS is often used as a measure of a post-medical procedure outcome, as a guide to the benefit of a treatment of interest, and/or as an important risk factor for adverse events, hospital readmission, and mortality [11–13]. Therefore, understanding hospital LOS variability across various patients’ clinical and socio-demographic characteristics and hospitals’ characteristics, such as geographic region and hospital sizes, is always an important public health focus [9, 14–22].

Inpatient hospital LOS is the number of nights spent in hospital, calculated from the day of admission to the day of discharge [23]. This type of data can be treated as count data, and count data values are usually nonnegative with a typically right-skewed distribution, often exhibiting excessive zeros and overdispersion [17, 24, 25]. Different analytic strategies have been used for modeling hospital LOS. However, the best way to model LOS and other right skewed data has been debated in the literature. Literature review showed that non-transformed or logarithm-transformed count outcome variable are often modeled with linear regression [26–28]. Linear regression is usually employed for continuous, normally, or approximately normally distributed outcomes. LOS data rarely adheres to these assumptions. Studies conducted to compare analyses of logarithm-transformed count outcome variables have reported several issues that might arise with such transformations, including zero values not considered, predicted meaningless negative values for the outcome variable, uninterpretable and biased parameter estimates, and inconsistent inferences about important policy parameters [29, 30]. Gardner et al. [31] showed that when the mean of the count outcome variable is small, linear regression produces biased standard errors and hence biased significance tests. Using simulation study, O’Hara [32] found that the log-transformations of count data often used to satisfy parametric test assumptions perform poorly, except when the dispersion was small, and the mean counts were large. When the mean count is very small and zero is the most common value in the data set, the normalization with log transformation will not work and the mode will always be at the lowest value [33]. Bryk et al. [34] stated, that there are important cases for which the assumption of linearity and normality are not realistic, and no transformation can make them so. An alternative approach that has been used as a solution to handle the non-normality of LOS outcome variable by researchers is to dichotomize LOS and use logistic regression to predict the LOS [35]. Dichotomizing count outcome variable lead to loss of information. Based on simulated and empirical data analyses, Sroka [36] concluded that more precise odds ratios estimates can be obtained using count regression models with log-odds link function. In summary, using linear regression models with or without logarithmic transformation of a count outcome variable, or logistic regression models on a dichotomized count outcome variable are subject to criticism for their inadequacy in modeling this type of data. This can lead to biased parameter estimates; prediction of meaningless negative values; and the loss of precision of inferences and important information about the underlying counts.

Common statistical methods for analysis of count data are Poisson, negative binomial (NB), zero-inflated Poisson (ZIP), and zero-inflated negative binomial (ZINB) regressions [24, 37–40]. The results from the existing research evaluating the performance of regression models for count data are conflicting regarding which model is preferred. Lambert (1992) compared ZIP to NB regression models in an experimental study concerning soldering defects on printing wiring boards where 81% of the board areas had 0 defects. He found that ZIP was better than the NB model in terms of prediction accuracy [37]. Greene (1994) compared Poisson, NB, ZIP, and ZINB models on a consumer loan behavior empirical data characterized with overdispersion and zero-inflation. In the analysis the author found that the NB model was superior to the ZIP model and the ZIP model was superior to the Poisson model in terms of model fit [41]. Slymen et al. (2006) compared Poisson, overdispersed Poisson, NB, ZIP and ZINB regression models in assessing predictors of vigorous physical activity among Latina women using data with 82% zeros in the outcome variable. They reported a little difference in ZIP and ZINB models' fits, however, overall, the ZIP model fitted best [42]. In overdispersed and zero-inflated data of the number of incidents involving human papillomavirus infection, Lee et al. (2012) found that ZIP, followed by NB, and ZINB had the smallest Akaike’s information criteria (AIC); and ZIP model showed the same results as the NB model regarding the covariates at a 0. 05 significance level. In addition, ZINB model did not always converged [43]. Tuzen et al. (2018) examined the performance in terms of fit of Poisson, NB, ZIP, ZINB, Poisson Hurdle and NB Hurdle models under various outliers and zero-inflation scenarios of simulated data and found that ZINB and NB Hurdle were superior to Poisson, NB, and ZIP models. They also reported that in some scenarios, the NB model outperformed all models in the presence of outliers and/or excess zeros [44]. Tlhaloganyang et al. [45] compared NB with ZIP and ZINB models using different real datasets characterized by overdispersion and zero-inflation. The authors found that NB provided a superior fit in all datasets [45].

Based on the reviewed literature, the question remains open to whether the different results in terms of model fit may arise from the different proportion of zeros, overdispersion, and sample size of the datasets used in these studies. In this study we had two objectives. The first objective was to compare the performance of Poisson, NB, ZIP, and ZINB regression models in simulation study. The second objective was to compare the performance of Poisson, NB, ZIP, and ZINB regression models using real life hospital data in assessing the effect of age, sex, health insurance status, and type of hospital admission on the hospital LOS. This research added to previous studies by including additional experimental scenarios, such as varying sample sizes, larger dispersion levels, various proportions of zero in the outcome variable, and data generated using Poisson and ZIP distributions, along with NB and ZINB distributions.

## Methods

### Overview of count data regression models

#### Poisson model

The most widely used and the most basic model that explicitly considers the nonnegative integer-valued aspect of the count outcome variable is the Poisson regression model [46]. Let ${Y}_{i}, i=1,\dots ,n$, be random variables for the number of occurrences of the event of interest and its realizations ${y}_{i}=0, 1, 2\dots$. Let ${{\varvec{X}}}_{{\varvec{i}}}^{\boldsymbol{^{\prime}}}=\left({X}_{1i}, \dots , {X}_{ki}\right)$ be a k-dimensional random vector of predictors and its realization ${{\varvec{x}}}_{{\varvec{i}}}^{\boldsymbol{^{\prime}}}=\left({x}_{1i}, \dots , {x}_{ki}\right), i=1,\dots ,n$. Poisson regression assumes that the dependent variable *Y*_*i*_, given ${{\varvec{X}}}_{{\varvec{i}}}={{\varvec{x}}}_{{\varvec{i}}}$ i = 1, …, n, is independently Poisson-distributed with:

1 $$P\left({{Y}_{i}=y}_{i}|{{{\varvec{X}}}_{{\varvec{i}}}={\varvec{x}}}_{{\varvec{i}}}\right)=\frac{{e}^{-{\mu }_{i}}{\mu }_{i}^{{y}_{i}}}{{y}_{i}!}, {y}_{i}=0, 1, 2, \dots$$

and the mean parameter (i.e., the mean number of events per period) is given by:

2 $${\mu }_{i}={e}^{{{\varvec{x}}}_{{\varvec{i}}}^{\boldsymbol{^{\prime}}}\beta }$$

where $\beta$ is a column vector of parameters.

In the Poisson regression model the conditional mean and the conditional variance of *Y*_*i*_ are equal (equidispersion):

3 $${E\left({{Y}_{i}|{{\varvec{X}}}_{{\varvec{i}}}={\varvec{x}}}_{i}\right)=V\left({{Y}_{i}|{{\varvec{X}}}_{{\varvec{i}}}={\varvec{x}}}_{i}\right)=\mu }_{i}$$

Poisson regression model is also called log-linear model because the logarithm of the conditional mean is linear in the parameters:

4 $${\mathrm{ln}(E\left({{Y}_{i}|{{\varvec{X}}}_{{\varvec{i}}}={\varvec{x}}}_{i}\right))=\mathrm{ln}(\mu }_{i})={{\varvec{x}}}_{i}^{^{\prime}}\beta$$

The marginal effect of a predictor variable ${X}_{j}$ is given by:

5 $$\frac{\partial E\left({Y}_{i}|{{\varvec{X}}}_{{\varvec{i}}}={{\varvec{x}}}_{{\varvec{i}}}\right)}{\partial {x}_{ji}}={{\beta }_{j}e}^{{{\varvec{x}}}_{{\varvec{i}}}^{\boldsymbol{^{\prime}}}\beta }={\beta }_{j}E\left({{Y}_{i}|{{\varvec{X}}}_{{\varvec{i}}}={\varvec{x}}}_{i}\right)$$

and the interpretation of this effect is that a one-unit change in the j^th^ predictor leads to a ${\beta }_{j}$ change in the conditional mean $E\left({{Y}_{i}|{{\varvec{X}}}_{{\varvec{i}}}={\varvec{x}}}_{i}\right)$

Real-life count data often exhibit two (related) characteristics: overdispersion and zero-inflation. Overdispersion refers to an excess of variability in the data (i.e., the variance exceeds the mean), while zero-inflation refers to an excess of zeros [39, 47]. In the presence of overdispersion, the Poisson regression model is not adequate and can lead to biased parameter estimates and unreliable standard errors estimates [38, 39]. The most commonly used model that accounts for overdispersion is the negative binomial model.

#### Negative binomial model

The Poisson regression model can be generalized by introducing an unobserved heterogeneity term for observation *i*. The subjects are assumed to differ randomly in a manner that is not fully accounted for by the observed covariates. This is formulated as:

6 $${E\left({{Y}_{i}|{{\varvec{X}}}_{{\varvec{i}}}={\varvec{x}}}_{i},{\tau }_{i}\right)=\mu }_{i}{\tau }_{i}={e}^{{{\varvec{x}}}_{{\varvec{i}}}^{\boldsymbol{^{\prime}}}\beta +{\varepsilon }_{i}}$$

where the unobserved heterogeneity term ${\tau }_{i}={e}^{{\varepsilon }_{i}}$ is independent of the vector of predictor variables *x*_*i*_. Then the conditional distribution of *Y*_*i*_ on ${{\varvec{X}}}_{{\varvec{i}}}={{\varvec{x}}}_{{\varvec{i}}}$ is Poisson with conditional mean and conditional variance ${\mu }_{i}{\tau }_{i}$:

7 $$f\left({{Y}_{i}=y}_{i}|{{{\varvec{X}}}_{{\varvec{i}}}={\varvec{x}}}_{{\varvec{i}}},{\tau }_{i}\right)=\frac{{e}^{-{\mu }_{i}{\tau }_{i}}{({\mu }_{i}{\tau }_{i})}^{{y}_{i}}}{{y}_{i}!}, {y}_{i}=0, 1, 2, \dots$$

The negative binomial distribution is derived as a gamma mixture of Poisson random variables [39, 48–50]. By letting $g\left({\tau }_{i}\right)$ be the probability density function of ${\tau }_{i}$, the distribution $f\left({{Y}_{i}=y}_{i}|{{\varvec{X}}}_{{\varvec{i}}}={{\varvec{x}}}_{{\varvec{i}}} \right)$ is obtained by integrating $f\left({{Y}_{i}=y}_{i}|{{{\varvec{X}}}_{{\varvec{i}}}={\varvec{x}}}_{{\varvec{i}}},{\tau }_{i}\right)$ with respect to ${\tau }_{i}.$ The analytical solution of the integral exists if ${\tau }_{i}$ is gamma distributed and this solution is the NB distribution. Specifically, it is necessary to assume that $E\left({\tau }_{i}\right)=1,$ and then ${\tau }_{i}$ follows gamma *(θ, θ)* distribution with $E\left({\tau }_{i}\right)=1$ and $V\left({\tau }_{i}\right)=\frac{1}{\theta }.$ It can be shown, that the NB distribution can be written as:

8 $$f\left({{Y}_{i}=y}_{i}|{{{\varvec{X}}}_{{\varvec{i}}}={\varvec{x}}}_{{\varvec{i}}}\right)=\frac{\Gamma \left({y}_{i}+{\alpha }^{-1}\right)}{{y}_{i}!\Gamma \left({\alpha }^{-1}\right)}{\left(\frac{{\alpha }^{-1}}{{\alpha }^{-1}+{\mu }_{i}}\right)}^{{\alpha }^{-1}}{\left(\frac{{\upmu }_{\mathrm{i}}}{{\alpha }^{-1}+{\mu }_{i}}\right)}^{{\mathrm{y}}_{\mathrm{i}}}, {y}_{i}=0, 1, 2, \dots$$

where ${\alpha }^{-1}=\theta$ and $\theta >0$ is the gamma scale parameter.

The NB conditional mean and conditional variance of the outcome variable ${y}_{i}$ are given by:

9 $${E\left({{Y}_{i}|{{\varvec{X}}}_{{\varvec{i}}}={\varvec{x}}}_{i}\right)=\mu }_{i}$$

10 $$V\left({{Y}_{i}|{{\varvec{X}}}_{{\varvec{i}}}={\varvec{x}}}_{i}\right)={\mu }_{i}(1+\alpha {\mu }_{i})>E\left({{y}_{i}|x}_{i}\right)$$

The parameter $\alpha$ is defined as the dispersion parameter. As $\alpha$ approaches zero (i.e., the gamma scale parameter $\theta$ approaches infinity), $V\left({{Y}_{i}|{{\varvec{X}}}_{{\varvec{i}}}={\varvec{x}}}_{i}\right)$ decreases to ${\mu }_{i}$=$E\left({{Y}_{i}|{{\varvec{X}}}_{{\varvec{i}}}={\varvec{x}}}_{i}\right),$ and the NB distribution approaches the Poisson distribution. Thus, the Poisson regression model is nested within the NB regression model.

#### Zero-inflated count models

Zero-inflated count models provide a way to both model the excess zeros and the overdispersion (He et al. 2014) [51]. In particular, there are two possible data generation processes for the number of occurrences of the event of interest ${y}_{i}$ for each observation i = 1,…,n and the result of a Bernoulli trial is used to determine which of the two to use. For observation *i*, Process 1 is chosen with probability ${\varphi }_{i}$ and Process 2 with probability $1-{\varphi }_{i}$. Process 1 generates only zero counts (“structural” zeros). Process 2 generates counts from either a Poisson model [37] or a NB model [41]. $P\left({Y}_{i}={y}_{i}|{{{\varvec{X}}}_{{\varvec{i}}}={\varvec{x}}}_{{\varvec{i}}}\right)$ can be described as follows:

11 $$P\left({Y}_{i}={y}_{i}|{{{\varvec{X}}}_{{\varvec{i}}}={\varvec{x}}}_{i}\right)=\left\{\begin{array}{ll}{\varphi }_{i}+(1-{\varphi }_{i})g(0)& {I}_{({y}_{i}=0)}\\ (1-{\varphi }_{i})\mathrm{g}({y}_{i})& {I}_{({y}_{i}>0)}\end{array}\right.$$

where $\mathrm{g}({y}_{i})$ follows either Poisson or NB distributions, defined in (1) and (8), respectively, and therefore the zero-inflated count models are called either zero-inflated Poisson (ZIP) or zero-inflated negative binomial (ZINB) regression models, respectively.

Further, if ${\varphi }_{i}$ depends on the characteristics of observation *i*, then ${\varphi }_{i}={F}_{i}=F({z}_{i}^{^{\prime}}\gamma )$, where ${z}_{i}$ is a (q + 1)-dimensional vector of zero-inflated covariates and $\gamma$ is a (q + 1)-dimensional vector of zero-inflated regression coefficients to be estimated. The function *F* is called zero-inflated link function.

In the case of the ZIP regression model, the conditional expectation and the conditional variance of the outcome variable ${Y}_{i}$ are given by:

12 $${E\left({{Y}_{i}|{{\varvec{X}}}_{{\varvec{i}}}={\varvec{x}}}_{i}, {z}_{i}\right)=\mu }_{i}(1-{F}_{i})$$

13 $$V\left({{Y}_{i}|{{\varvec{X}}}_{{\varvec{i}}}={\varvec{x}}}_{i}, {z}_{i}\right)=E\left({{Y}_{i}|{{\varvec{X}}}_{{\varvec{i}}}={\varvec{x}}}_{i}, {z}_{i}\right)(1+{F}_{i}{\mu }_{i})>E\left({{Y}_{i}|{{\varvec{X}}}_{{\varvec{i}}}={\varvec{x}}}_{i}, {z}_{i}\right)$$

Since $V\left({{Y}_{i}|{{\varvec{X}}}_{{\varvec{i}}}={\varvec{x}}}_{{\varvec{i}}}, {z}_{i}\right)>E\left({{Y}_{i}|{{\varvec{X}}}_{{\varvec{i}}}={\varvec{x}}}_{{\varvec{i}}}, {z}_{i}\right)$, ZIP model exhibits overdispersion as well.

In the case of ZINB regression model, the conditional expectation and the conditional variance of the outcome variable ${Y}_{i}$ are given by:

14 $${E\left({{Y}_{i}|{{\varvec{X}}}_{{\varvec{i}}}={\varvec{x}}}_{i}, {z}_{i}\right)=\mu }_{i}(1-{F}_{i})$$

15 $$V\left({{Y}_{i}|{{\varvec{X}}}_{{\varvec{i}}}={\varvec{x}}}_{i}, {z}_{i}\right)=E\left({{{\varvec{Y}}}_{{\varvec{i}}}|{{\varvec{X}}}_{{\varvec{i}}}={\varvec{x}}}_{{\varvec{i}}}, {z}_{i}\right)[1+{(\alpha +F}_{i}{)\mu }_{i}]$$

Since $V\left({{{\varvec{Y}}}_{{\varvec{i}}}|{{\varvec{X}}}_{{\varvec{i}}}={\varvec{x}}}_{{\varvec{i}}}, {z}_{i}\right)>E\left({{{\varvec{Y}}}_{{\varvec{i}}}|{{\varvec{X}}}_{{\varvec{i}}}={\varvec{x}}}_{{\varvec{i}}}, {z}_{i}\right)$, the ZINB model like ZIP model exhibits overdispersion as well. Just as the NB distribution converges to the Poisson distribution as $\alpha$ approaches zero, the ZINB distribution converges to the ZIP distribution as $\alpha$ approaches zero.

#### Generalized linear models

Poisson, NB, ZIP, and ZINB are all part of the Generalized Linear Models (GLMs). The term GLM refers to a large class of models first introduced by Nelder and Wedderburn [52] and further developed and explained by McCullagh and Nelder [53]. GLMs extend standard linear regression models to encompass non-normal response distributions and possibly nonlinear functions of the mean [40]. The ordinary linear regression model uses linearity to describe the relationship between the mean of the response variable and a set of explanatory variables, with inference assuming that the response distribution is normal [40]. GLMs have three components: 1) A random component, that specifies the response variable *Y*_*i,*_ for the *i*^th^ observation and its probability distribution. 2) A inear component, ${\eta }_{i}={{\varvec{X}}}_{i}^{^{\prime}}\beta$, where $\beta$ is a column vector of parameters and ${{\varvec{X}}}_{i}$ is a column vector of predictors for the *i*th observation. 3) A monotonic differentiable link function *g(.)* describing how the expected value of variable *Y*_*i*_ is related to the linear predictor ${\eta }_{i}$, ${g\left[E\left(Yi\right)\right]=g({\mu }_{i}) = {\varvec{X}}}_{i}^{^{\prime}}\beta$, [40]. The response variable *Y*_*i*_ are independent for *i* = 1, 2, and have a probability distribution for an exponential family. This implies that the variance of the response variable *Y*_*i*_ depends on the mean ${\mu }_{i}$ through a variance function *V:* $var\left({Y}_{i}\right)=\frac{\phi V\left({\mu }_{i}\right)}{{\omega }_{i}},$ where $\phi$ is a constant, known as dispersion parameter, and ${\omega }_{i}$ is a known weight for each observation. The link function *g* for Poisson, NB, ZIP and ZINB regression models is log (${\eta }_{i}=\mathrm{log}({\mu }_{i})$). The binary link function *h* for the model of the probability of a zero count in the case of ZIP and ZINB regression models*,* is one of the logit, probit, or complementary log–log.

### Simulation study

#### Dataset generation

Several datasets with one dependent variable *y* and two predictor variables *x*_*1*_ and *x*_*2*_ were generated from the following four distributions: Poisson, NB, ZIP, and ZINB. Variable *x*_*1*_ was continuous and generated from a normal distribution with mean *µ* = *57.3* and a variance *σ*^*2*^ = *306.25* representing the distribution of variable age observed in the Medical Information Mart for Intensive Care (MIMIC-III) dataset for patients with an asthma diagnosis [54–56]. The binary variable *x*_*2*_ was generated from Bernoulli distribution with probability of success *p* = *0.43*, representing the distribution of variable sex in the MIMIC-III dataset for patients with an asthma diagnosis. The values of the population regression coefficient *β*_*0*_*, β*_*1*_, and *β*_*2*_ were pre-specified and obtained by fitting a NB regression model for the outcome variable hospital LOS in the same MIMIC-III dataset. For each of the simulated data under Poisson, NB, ZIP, and ZINB distributions, four different sample size scenarios were considered (50, 200, 600, and 1000). In the cases of count data generated with NB distribution or ZINB distribution different levels of dispersion (0.01, 1, 5, and 10) were considered under each of the sample size simulation scenarios. In the cases of count data generated with ZIP distribution or ZINB distribution, different proportions of structural zero (0.1, 0.3, 0.5, and 0.7) were considered under each of the sample size simulation scenario and under each of the dispersion levels simulation scenarios for data generated under ZINB distribution. To minimize the impact of simulation error, each scenario was repeated 1000 times. A summary of the simulation scenarios considered in the study is shown in Table 1.

**Table 1:** Simulation scenarios considered in the simulation study

Distribution	Sample size, n	Proportion of Zeros	Level of Dispersion
Poisson	50	0.1	0.01
NB	200	0.3	1
ZIP	600	0.5	5
ZINB	1000	0.70	10

#### Models evaluation

Poisson, NB, ZIP, and ZINB regression models with dependent variable *y* and independent variables *x*_*1*_ and *x*_*2*_ were fitted on the generated data under each of the simulation scenarios and replications using the maximum likelihood estimation (MLE) method [57]. The Quasi-Newton optimization technique was used to maximize the likelihood functions to obtain the regression models' estimates. To assess the performance of the four different models under each simulation scenario, we first calculated the models’ convergence rates. When the MLE procedure converged, it means it found a unique set of values for each parameter, the combination of which returned the highest likelihood value of all parameter values examined [58]. For the converged models, we extracted the widely-used Akaike’s Information Criteria (AIC), a model selection criterion developed by Hirotsugu Akaike [59].We also used the Bayesian information criteria (BIC) (also Schwarz criterion, SBC, SBIC) first formulated by Gideon Schwarz [60]. Smaller values of these criteria indicate a better model fit. In addition to the AIC and BIC statistics, we calculated the mean absolute error (MAE) for the *E(y*_*i*_*|x*_*i*_*),* defined as $MAE=\frac{{\sum }_{i=1}^{n}|E\left({y}_{i}|{x}_{i}\right)-E({\widehat{y}}_{i}|{x}_{i})|}{n},$ where *n* is the sample size. The AIC, BIC, and MAE were averaged over the 1000 replications in each of the simulation scenario. All simulations and statistical analyses were conducted using Statistical Analysis System (SAS) 9.4 (SAS Institute, Inc., 2015).

### Empirical study

#### Data description

In the empirical study we used data from the Medical Information Mart for Intensive Care (MIMIC-III) [54–56]. MIMIC-III is a large, single-center database comprising information relating to patients admitted at the Beth Israel Deaconess Medical Center in Boston, Massachusetts. Agreement for data use was obtained. For the purposes of our study, we extracted patients with International Classification of Diseases, ninth revision (ICD-9) Code 49,390, which is related to the diagnosis of asthma. The resultant dataset consisted of 2,195 hospital patient admission records.

In our study, the outcome variable of interest was hospital LOS, calculated as the difference in days between the date of admission and the date of discharge. The predictor variables considered in the regression analyses were age, sex, patient health insurance and type of admission. Age was measured in years. Sex was a categorical variable with two levels: male and female. Patient health insurance was a categorical variable with 5 levels: government, Medicaid, Medicare, private, and self-pay. Admission type was a categorical variable with 3 levels: elective, emergency, and urgent.

#### Statistical analysis

First, we conducted descriptive statistical analysis to summarize and describe the study data. Frequencies and percentages were used to describe categorical variables and means, and standard deviations were used to describe continuous variables. The distribution of the count outcome variable LOS was visually examined using histogram. In addition, we calculated the variance and the mean of the outcome variable LOS to highlight potential Poisson distribution violations and overdispersion in the data. Poisson, NB, ZIP, and ZINB regression models were fitted for LOS on the predictor variables age, sex, health insurance, and admission type. The Pearson dispersion statistic, calculated by dividing the model's Pearson Chi-square statistic by the corresponding degrees of freedom, was used as a criterion for assessing model's misspecification or an overdispersed response variable. When the resultant value is greater than one, the model is considered to be overdispersed. AIC and BIC were used to compare the models. Furthermore, the models estimated coefficients, standard errors and their significance where examined, giving special attention to the difference in findings and conclusions across the models. All regression models were fitted using PROC COUNTREG and PROC GENMOD in SAS 9.4 (SAS Institute, 2015). Statistical testing was two-sided and performed at a significance (α) level of 0.05.

## Results

### Simulation study

#### Data generated with poisson regression model

Table 2 shows the convergence rates of Poisson, NB, ZIP, and ZINB regression models for simulated data in four different sample size scenarios (*n* = 50, 200, 600, 1000). NB regression convergence rate was between 53.5% and 58.7% with the largest convergence rate achieved in simulated samples of size 1000 (Table 2). The convergence rate for ZINB increased from 90.4% to 94.2% as the sample size increased from 50 to 1000. Both Poisson and ZIP regression models had 100% convergence rate in all simulation scenarios.

**Table 2:** Convergence rates of regression models fitted on data generated with Poisson regression model

Sample Size	Poisson	NB	ZIP	ZINB
50	100.0%	53.5%	100.0%	90.4%
200	100.0%	53.6%	100.0%	93.4%
600	100.0%	54.9%	100.0%	93.5%
1000	100.0%	58.7%	100.0%	94.2%

Table 3 shows the averaged AIC and BIC statistics produced for Poisson, NB, ZIP, and ZINB regression models across all replications under each of the four sample size simulation scenarios. Poisson regression model (true model) resulted with the lowest mean AIC and BIC values, followed by the NB regression model. The difference in mean AIC and mean BIC values between the fitted Poisson and the fitted NB and ZINB regression models increased as the sample size increased.

**Table 3:** AIC and BIC of regression models fitted on data generated with Poisson regression model

	AIC				BIC			
Sample Size	Poisson	NB	ZIP	ZINB	Poisson	NB	ZIP	ZINB
50	260.88	266.12	266.88	267.92	266.6152	273.3131	278.3513	280.1757
200	1032.82	1042.64	1038.82	1040.18	1042.7200	1054.9800	1058.6100	1061.4600
600	3092.07	3107.19	3098.07	3100.35	3105.2600	3123.6300	3124.4500	3128.8100
1000	5151.60	5173.91	5157.60	5160.71	5166.3200	5192.7200	5187.0400	5192.5700

Table 4 shows the MAE values of the predicted counts by the fitted Poisson, NB, ZIP, and ZINB regression models across all replications under each of the four sample size simulation scenarios. Poisson regression model (true model) resulted with the lowest MAE followed by the ZIP, ZINB and NB regression models.

**Table 4:** MAE values of regression models fitted on data generated with Poisson regression model

Sample Size	Poisson	NB	ZIP	ZINB
50	0.6201	0.6158	0.6202	0.6202
200	0.3039	0.3055	0.3055	0.3045
600	0.1727	0.1764	0.1736	0.1736
1000	0.1401	0.1420	0.1402	0.1402

#### Data generated with NB regression model

In this section we describe the analysis of data generated with NB regression model in sixteen different simulation scenarios with varying sample sizes (*n* = 50, 200, 600, 1000) and magnitudes of dispersion (0.01, 1, 5, 10). Table 5 shows the convergence rates of Poisson, NB, ZIP, and ZINB regression models. At very low levels of overdispersion (0.01) the NB regression model's (true model) convergence rate ranged from 64.8% in the scenario with the smallest sample size to 94% in the scenario with the largest sample size. Similarly, in the same scenario of low level of overdispersion the ZINB regression model did not achieve 100% convergence rate. Overall, the ZINB regression model’s convergence rate was slightly better than the convergence rate of the NB model, ranging between 91% and 99.6% with the increase of the sample size. Poisson and ZIP regression models converged 100% in all simulation scenarios. In the rest of the scenarios of level of dispersion (1, 5, and 10) and all the sample sizes (*n* = 50,200,600 and 1000), all the models achieved 100% convergence rate.

**Table 5:** Convergence rates of regression models fitted on data generated with NB regression model

Sample Size	Dispersion	Poisson	NB	ZIP	ZINB
50	0.01	100.0%	64.8%	100.0%	91.0%
	1	100.0%	100.0%	100.0%	100.0%
	5	100.0%	100.0%	100.0%	100.0%
	10	100.0%	100.0%	100.0%	100.0%
200	0.01	100.0%	81.2%	100.0%	97.3%
	1	100.0%	100.0%	100.0%	100.0%
	5	100.0%	100.0%	100.0%	100.0%
	10	100.0%	100.0%	100.0%	100.0%
600	0.01	100.0%	90.5%	100.0%	99.5%
	1	100.0%	100.0%	100.0%	100.0%
	5	100.0%	100.0%	100.0%	100.0%
	10	100.0%	100.0%	100.0%	100.0%
1000	0.01	100.0%	94.0%	100.0%	99.6%
	1	100.0%	100.0%	100.0%	100.0%
	5	100.0%	100.0%	100.0%	100.0%
	10	100.0%	100.0%	100.0%	100.0%

Table 6 displays the averaged AIC and BIC model fit statistics for Poisson, NB, ZIP, and ZINB regression models fitted on data generated with NB regression model with different magnitudes of dispersion and sample sizes. In the simulation scenario with nearly nonexistent overdispersion level (0.01), the Poisson regression model had the lowest AIC and BIC values regardless of the sample size. In all other simulation scenarios, the NB regression model (true model) had the lowest AIC and BIC values. The model fit of the NB and ZINB models improved as the level of dispersion increased; conversely, the model fit of Poisson and ZIP regression modes decreased as the dispersion level increased.

**Table 6:** AIC and BIC of regression models fitted on data generated with NB regression model

		AIC				BIC			
Sample Size	Dispersion	Poisson	NB	ZIP	ZINB	Poisson	NB	ZIP	ZINB
50	0.01	265.51	270.12	271.51	272.78	271.24	277.42	282.98	285.40
	1	635.25	341.14	634.90	346.91	640.98	348.79	646.37	360.29
	5	1346.69	276.80	858.14	282.22	1352.43	284.45	869.61	295.60
	10	1766.50	216.93	956.54	221.37	1772.24	224.58	968.01	234.75
200	0.01	1051.89	1057.24	1057.89	1058.58	1061.79	1069.85	1077.68	1081.01
	1	2618.54	1352.80	2612.87	1358.58	2628.44	1365.99	2632.66	1381.66
	5	5908.13	1095.27	3712.59	1100.91	5918.03	1108.46	3732.38	1124.01
	10	7900.24	862.34	4125.06	866.21	7910.14	875.53	4144.85	889.29
600	0.01	3150.60	3153.55	3156.60	3155.11	3163.79	3170.49	3182.98	3185.66
	1	7943.23	4057.43	7671.57	4063.25	7956.42	4075.02	7697.96	4094.03
	5	17,923.15	3274.81	11,317.07	3280.08	17,936.34	3292.41	11,343.45	3310.85
	10	24,241.73	2571.64	12,792.10	2575.34	24,254.92	2589.23	12,818.48	2606.12
1000	0.01	5250.77	5251.31	5256.77	5253.24	5265.49	5270.52	5286.21	5287.51
	1	13,213.43	6753.31	12,743.07	6758.97	13,228.15	6772.94	12,772.52	6793.33
	5	29,964.16	5455.19	18,966.36	5460.53	29,978.88	5474.82	18,995.81	5494.89
	10	40,391.38	4273.81	21,385.72	4277.43	40,406.10	4293.44	21,415.17	4311.78

MAE values of the predicted counts with Poisson, NB, ZIP, and ZINB regression models fitted on data generated with NB regression model with different magnitude of dispersion and sample sizes are shown in Table 7. In the simulation scenario with the smallest sample size (*n* = 50), the Poisson regression model resulted with the smallest MAE regardless of the dispersion level. When the sample size increased to (*n* = 200) and at very small levels of overdispersion (0.01), both Poisson and ZIP regression models produced the smallest MAE, followed by NB regression model. When the dispersion level was 10 and sample seizes were greater than 200, NB, followed by ZIP regression model produced the lowest MAE. In the scenarios with sample sizes greater than 200 the true model (i.e., NB regression model) produced the smallest MAE regardless of the level of dispersion.

**Table 7:** MAE values of regression models fitted on data generated with NB regression model

Sample Size	Dispersion	Poisson	NB	ZIP	ZINB
50	0.01	0.6457	0.6501	0.6501	0.6477
	1	2.0766	2.0981	2.0808	2.1070
	5	4.3471	4.7621	4.4190	4.7984
	10	6.0011	7.4481	6.2916	7.5888
200	0.01	0.3263	0.3297	0.3263	0.3267
	1	1.0397	1.0384	1.0412	1.0402
	5	2.2255	2.2529	2.2250	2.2651
	10	3.2408	3.0910	3.1349	3.3494
600	0.01	0.1836	0.1828	0.1836	0.1839
	1	0.6050	0.6020	0.6057	0.6024
	5	1.2813	1.2810	1.2853	1.2935
	10	1.8427	1.8309	1.8340	1.8844
1000	0.01	0.1430	0.1427	0.1430	0.1429
	1	0.4701	0.4677	0.4696	0.4683
	5	0.9983	0.9959	0.9972	1.0025
	10	1.4665	1.4614	1.4629	1.4951

#### Data generated with ZIP regression model

Table 8 shows the convergence rates of Poisson, NB, ZIP, and ZINB regression models fitted on data generated with a ZIP distribution in simulation scenarios with different levels of structural zeros and sample sizes. Poisson and ZIP regression models achieved 100% convergence rate across all the simulation scenarios. NB regression model achieved 98.4% and 99.9% convergence rate in data simulated with 10% proportion of structural zeros and sample sizes *n* = 50 and *n* = 200, respectively, and achieved 100% convergence rate in all the combination of scenarios for sample sizes and proportion of structural zeros greater than 30%. ZINB regression model convergence rate varied between 94.4% to 99.4% across all the simulation scenarios.

**Table 8:** Convergence rates of regression models fitted on data generated with ZIP regression model

Sample Size	Proportion of Zeros	Poisson	NB	ZIP	ZINB
50	0.1	100.0%	98.4%	100.0%	96.8%
	0.3	100.0%	100.0%	100.0%	97.9%
	0.5	100.0%	100.0%	100.0%	96.0%
	0.7	100.0%	100.0%	100.0%	95.0%
200	0.1	100.0%	99.9%	100.0%	95.6%
	0.3	100.0%	100.0%	100.0%	98.3%
	0.5	100.0%	100.0%	100.0%	96.7%
	0.7	100.0%	100.0%	100.0%	94.4%
600	0.1	100.0%	100.0%	100.0%	95.0%
	0.3	100.0%	100.0%	100.0%	97.4%
	0.5	100.0%	100.0%	100.0%	97.9%
	0.7	100.0%	100.0%	100.0%	94.9%
1000	0.1	100.0%	100.0%	100.0%	95.2%
	0.3	100.0%	100.0%	100.0%	99.4%
	0.5	100.0%	100.0%	100.0%	96.2%
	0.7	100.0%	100.0%	100.0%	95.0%

Table 9 shows the AIC and BIC fit statistics values for the fitted Poisson, NB, ZIP, and ZINB regression models on data generated with ZIP regression model. When the proportion of structural zeros was 10%, the ZINB regression model had the smallest AIC and BIC values rather than the true ZIP regression model. In simulation scenarios with a higher proportion of structural zeros in the data (30%, 50%, 70%), the true ZIP regression model had the smallest AIC and BIC values followed by the ZINB regression model. In addition, the Poisson regression model produced the largest AIC and BIC values in all the scenarios. Another finding was that Poisson models fit became worse as the proportion of structural zeros increased from 10 to 50%, then slightly improved when the proportion of structural zeros reached 70%; contrary to the rest of the models, where the fit considerably became better as the proportion of structural zeros increased.

**Table 9:** AIC and BIC of regression models fitted on data generated with ZIP regression model

		AIC				BIC			
Sample Size	Proportion of Zeros	Poisson	NB	ZIP	ZINB	Poisson	NB	ZIP	ZINB
50	0.1	330.55	309.147	325.14	294.95	334.992	315.629	335.50	306.43
	0.3	433.015	307.129	261.57	298.20	437.754	315.322	273.83	311.98
	0.5	475.166	256.639	204.79	235.84	479.329	263.507	215.14	244.69
	0.7	431.434	179.094	144.05	156.10	434.473	186.103	154.85	166.43
200	0.1	1316.84	1225.51	1314.41	1109.70	1329.63	1239.56	1338.93	1130.06
	0.3	1749.24	1215.96	970.82	1138.99	1756.33	1229.85	990.56	1160.73
	0.5	1936.33	1009.57	798.26	880.09	1942.94	1020.93	815.54	902.58
	0.7	1782.48	698.492	559.00	568.73	1793.98	714.057	580.31	590.12
600	0.1	3960.45	3674.16	3703.56	3219.10	3969.41	3690.25	3719.15	3256.71
	0.3	5250.21	3641.94	2902.96	3292.35	5267.11	3657.86	2926.41	3308.48
	0.5	5834.42	3016.79	2381.02	2516.09	5845.18	3032.01	2404.95	2553.38
	0.7	5387.17	2084.75	1664.94	1666.35	5410.3	2107.83	1695.38	1698.18
1000	0.1	6596.51	6120.87	5475.69	5298.91	6611.8	6140.44	5520.85	5324.98
	0.3	8756.95	6066.9	4846.20	5902.66	8782	6084.58	4866.44	5907.23
	0.5	9735.86	5026.48	3966.62	3995.33	9745.98	5047.67	3996.49	4027.83
	0.7	8987.2	3469.05	2768.75	2770.02	9004.26	3487.81	2799.32	2801.99

The MAE values of the predicted counts based on Poisson, NB, ZIP, and ZINB regression models fitted on data generated with the ZIP regression model in different simulation scenarios are shown in Table 10. ZIP model had the lowest MAE in the scenarios with the smallest sample size (*n* = 50) and proportion of structural zeros of 10% and 30%. Both ZIP and ZINB had the lowest MAEs in the scenario with the smallest sample size (*n* = 50) and 50% proportion of structural zeros. ZINB had the lowest MAE in the scenarios with sample sizes 200 and 1000 and 70% proportion of structural zeros. The ZIP model produced the lowest MAEs in scenarios with sample sizes 200 and 1000 and 10% proportion of structural zeros. When the sample size was 600 and the proportion of structural zeros was 30%, the ZINB had the highest MAE while Poisson, NB and ZIP had the same MAE. In all other scenarios, NB produced the lowest MAE.

**Table 10:** MAE values of regression models fitted on data generated with ZIP regression model

Sample Size	Proportion of Zeros	Poisson	NB	ZIP	ZINB
50	0.1	1.2051	1.2175	1.2041	1.2056
	0.3	3.1300	3.1366	3.1202	3.1296
	0.5	5.2026	5.2005	5.1954	5.1954
	0.7	7.2560	7.2425	7.2506	7.2345
200	0.1	1.0618	1.0624	1.0616	1.0638
	0.3	3.1064	3.1057	3.1061	3.1059
	0.5	5.1843	5.1827	5.1841	5.1791
	0.7	7.2264	7.2231	7.2261	7.2265
600	0.1	1.0350	1.0350	1.0348	1.0347
	0.3	3.1078	3.1078	3.1078	3.1104
	0.5	5.1784	5.1784	5.1786	5.1814
	0.7	7.2242	7.2238	7.2241	7.2256
1000	0.1	1.0299	1.0298	1.0297	1.0301
	0.3	3.0995	3.0994	3.0995	3.1000
	0.5	5.1630	5.1628	5.1630	5.1633
	0.7	7.2381	7.2378	7.2380	7.2381

#### Data generated with ZINB regression model

In this section, we present the results from the analysis of the data generated with the ZINB regression model on sixty-four different simulation scenarios with various proportions of zeros, magnitudes of dispersion, and sample sizes. Tables 11, 12, 13, 14 present the convergence rates of Poisson, NB, ZIP, and ZINB regression models fitted on the simulated data across different simulation scenarios. In the scenario where data were simulated with the smallest sample size (*n* = 50), the Poisson model achieved 100% convergence rate regardless of the magnitudes of dispersion or structural zero proportions (Table 11). The convergence rates for the other models were unstable across the simulation scenarios and varied between 94.6% and 100%. For instance, ZIP model convergence rate was 100% for dispersion levels 0.01, 1, and 5 regardless of the proportion of structural zeros; and varied between 98.9% and 99.9% in the scenario with the largest overdispersion (dispersion = 10). When the sample size was 50, the convergence rate of ZINB slightly reduced as the proportion of zeros increased. Only in the scenarios with dispersion levels of 1 and 5 and proportion of structural zeros of (10%, 30%, and 50%), the ZINB model achieved 100% convergence rate. However, the trend observed in the convergence rate of ZINB in scenarios with sample size 50 changed as the sample size became larger (Tables 11, 12, 13, 14). With the increase of the sample size all the models achieved 100% convergence rate in all simulation scenarios except for the NB and ZINB regression models, where in simulation scenarios with the smallest level of dispersion (dispersion = 0.01) the models’ convergence rates were slightly below 100%.

**Table 11:** Convergence rates of regression models fitted on data generated with ZINB regression model, *n* = 50

Dispersion	Proportion of Zeros	Poisson	NB	ZIP	ZINB
0.01	0.1	100.0%	98.3%	100.0%	97.4%
	0.3	100.0%	100.0%	100.0%	98.6%
	0.5	100.0%	100.0%	100.0%	97.3%
	0.7	100.0%	100.0%	100.0%	94.6%
1	0.1	100.0%	100.0%	100.0%	100.0%
	0.3	100.0%	100.0%	100.0%	100.0%
	0.5	100.0%	100.0%	100.0%	100.0%
	0.7	100.0%	100.0%	100.0%	99.9%
5	0.1	100.0%	100.0%	100.0%	100.0%
	0.3	100.0%	100.0%	100.0%	100.0%
	0.5	100.0%	100.0%	100.0%	100.0%
	0.7	100.0%	100.0%	99.8%	99.0%
10	0.1	100.0%	100.0%	99.9%	100.0%
	0.3	100.0%	100.0%	99.9%	99.8%
	0.5	100.0%	100.0%	99.9%	99.4%
	0.7	100.0%	100.0%	98.9%	95.0%

**Table 12:** Convergence rates of regression models fitted on data generated with ZINB regression model, *n* = 200

Dispersion	Proportion of Zeros	Poisson	NB	ZIP	ZINB
0.01	0.1	100.0%	99.8%	100.0%	98.8%
	0.3	100.0%	100.0%	100.0%	98.8%
	0.5	100.0%	100.0%	100.0%	98.6%
	0.7	100.0%	100.0%	100.0%	96.4%
1	0.1	100.0%	100.0%	100.0%	100.0%
	0.3	100.0%	100.0%	100.0%	100.0%
	0.5	100.0%	100.0%	100.0%	100.0%
	0.7	100.0%	100.0%	100.0%	99.9%
5	0.1	100.0%	100.0%	100.0%	100.0%
	0.3	100.0%	100.0%	100.0%	100.0%
	0.5	100.0%	100.0%	100.0%	100.0%
	0.7	100.0%	100.0%	100.0%	100.0%
10	0.1	100.0%	100.0%	100.0%	100.0%
	0.3	100.0%	100.0%	100.0%	100.0%
	0.5	100.0%	100.0%	100.0%	100.0%
	0.7	100.0%	100.0%	100.0%	100.0%

**Table 13:** Convergence rates of regression models fitted on data generated with ZINB regression model, *n* = 600

Dispersion	Proportion of Zeros	Poisson	NB	ZIP	ZINB
0.01	0.1	100.0%	99.8%	100.0%	99.3%
	0.3	100.0%	100.0%	100.0%	99.8%
	0.5	100.0%	100.0%	100.0%	98.9%
	0.7	100.0%	100.0%	100.0%	98.2%
1	0.1	100.0%	100.0%	100.0%	100.0%
	0.3	100.0%	100.0%	100.0%	100.0%
	0.5	100.0%	100.0%	100.0%	100.0%
	0.7	100.0%	100.0%	100.0%	100.0%
5	0.1	100.0%	100.0%	100.0%	100.0%
	0.3	100.0%	100.0%	100.0%	100.0%
	0.5	100.0%	100.0%	100.0%	100.0%
	0.7	100.0%	100.0%	100.0%	100.0%
10	0.1	100.0%	100.0%	100.0%	100.0%
	0.3	100.0%	100.0%	100.0%	100.0%
	0.5	100.0%	100.0%	100.0%	100.0%
	0.7	100.0%	100.0%	100.0%	100.0%

**Table 14:** Convergence rates of regression models fitted on data generated with ZINB regression model, *n* = 1000

Dispersion	Proportion of Zeros	Poisson	NB	ZIP	ZINB
0.01	0.1	100.0%	99.8%	100.0%	99.8%
	0.3	100.0%	100.0%	100.0%	99.9%
	0.5	100.0%	100.0%	100.0%	99.6%
	0.7	100.0%	100.0%	100.0%	99.1%
1	0.1	100.0%	100.0%	100.0%	100.0%
	0.3	100.0%	100.0%	100.0%	100.0%
	0.5	100.0%	100.0%	100.0%	100.0%
	0.7	100.0%	100.0%	100.0%	100.0%
5	0.1	100.0%	100.0%	100.0%	100.0%
	0.3	100.0%	100.0%	100.0%	100.0%
	0.5	100.0%	100.0%	100.0%	100.0%
	0.7	100.0%	100.0%	100.0%	100.0%
10	0.1	100.0%	100.0%	100.0%	100.0%
	0.3	100.0%	100.0%	100.0%	100.0%
	0.5	100.0%	100.0%	100.0%	100.0%
	0.7	100.0%	100.0%	100.0%	100.0%

The AIC and BIC fit statistics of Poisson, NB, ZIP, and ZINB regression models fitted on data generated with the ZINB regression model and with different magnitudes of dispersion, zero proportions, and sample sizes are displayed in Tables 15, 16, 17, 18. In the simulation scenarios with the smallest dispersion level of 0.01 and 10% structural zeros, ZINB produced the lowest AIC and BIC fit statistics values regardless of the sample size, except for the case with the smallest sample size, where NB model had the lowest AIC values. However, ZINB had the lowest BIC values across all scenarios. When the proportion of structural zeros was greater than 10% ZIP model produced the lowest AIC and BIC statistics in all simulation scenarios with a dispersion level of 0.01 regardless of the sample size. When the dispersion level reached 1, the regression model that produced the lowest AIC values was the ZINB model in nearly all the scenarios of different sample sizes and proportions of structural zeros, except for the scenario with sample size 50, where NB produced the lowest AIC values in scenarios with a proportion of structural zeros up to 50%. ZINB produced the lowest AIC in the simulation scenarios with proportion of structural zeros greater than 50% and dispersion equal to 1. Similarly, at level of dispersion equal to 1 the model that produced the lowest BIC was the NB model in all the simulation scenarios of proportion of structural zeros and a small sample size 50. When the sample size increased from 50 to 1000, the NB regression model produced the lowest BIC statistics in the simulation scenarios with proportion of structural zero below 50%. The ZINB regression model produced the lowest BIC fit statistics in the scenarios with a proportion of structural zeros exceeding 50%. In summary, based on AIC and BIC statistics NB regression model consistently produced the best fit in all simulation scenarios with a dispersion level exceeding 1 regardless of the proportion of structural zeros and sample size.

**Table 15:** AIC and BIC of regression models fitted on data generated with ZINB regression model, *n* = 50

		AIC				BIC			
Dispersion	Proportion of Zeros	Poisson	NB	ZIP	ZINB	Poisson	NB	ZIP	ZINB
0.01	0.1	334.99	310.68	328.98	314.61	340.73	318.27	340.46	311.05
	0.3	436.35	307.02	264.63	283.63	442.09	314.67	276.10	312.97
	0.5	476.33	256.23	207.07	230.73	482.07	263.88	218.54	249.45
	0.7	432.29	178.85	145.33	161.01	438.02	186.50	156.80	168.90
1	0.1	662.56	328.90	599.90	331.82	668.30	336.55	611.37	348.00
	0.3	684.04	288.86	451.98	289.87	689.77	296.51	463.45	306.57
	0.5	648.66	231.54	332.12	231.72	654.39	239.19	343.59	246.58
	0.7	516.19	159.30	206.90	158.71	521.93	166.95	218.37	172.42
5	0.1	1299.27	257.12	761.95	260.82	1305.00	264.77	773.42	275.50
	0.3	1152.80	212.91	573.34	216.00	1158.54	220.56	584.81	230.27
	0.5	955.49	165.12	396.99	167.50	961.23	172.77	408.46	181.53
	0.7	651.92	109.17	207.29	110.38	657.66	116.81	218.76	124.04
10	0.1	1642.30	199.36	822.90	202.74	1648.04	207.01	834.37	216.88
	0.3	1401.83	163.20	580.56	166.20	1407.56	170.84	592.04	180.44
	0.5	1103.92	125.17	380.16	128.91	1109.65	132.82	391.63	145.11
	0.7	757.17	83.21	178.00	85.78	762.91	90.84	189.47	103.55

**Table 16:** AIC and BIC of regression models fitted on data generated with ZINB regression model, *n* = 200

		AIC				BIC			
Dispersion	Proportion of Zeros	Poisson	NB	ZIP	ZINB	Poisson	NB	ZIP	ZINB
0.01	0.1	1333.77	1229.97	1332.39	1227.46	1343.66	1243.15	1352.18	1148.42
	0.3	1761.76	1215.33	984.06	1067.55	1771.66	1228.53	1003.85	1164.05
	0.5	1942.31	1008.44	806.91	852.92	1952.21	1021.64	826.70	910.07
	0.7	1786.95	697.77	564.36	645.10	1796.84	710.96	584.15	594.94
1	0.1	2742.24	1303.87	2488.31	1303.70	2752.14	1317.07	2508.10	1332.46
	0.3	2833.77	1140.65	1820.51	1133.77	2843.66	1153.84	1840.30	1166.58
	0.5	2705.47	914.06	1386.86	904.33	2715.37	927.26	1406.65	933.00
	0.7	2236.29	624.40	892.96	619.49	2246.18	637.60	912.75	636.68
5	0.1	5706.66	1016.90	3372.84	1020.43	5716.55	1030.09	3392.63	1045.04
	0.3	5072.56	839.10	2614.08	841.96	5082.46	852.29	2633.87	865.30
	0.5	4292.57	646.81	1890.03	649.02	4302.47	660.00	1909.82	672.33
	0.7	3147.23	429.58	1125.24	431.35	3157.12	442.78	1145.03	453.84
10	0.1	7469.74	794.21	3702.48	797.88	7479.63	807.40	3722.27	820.93
	0.3	6449.26	648.03	2850.89	651.08	6459.15	661.23	2870.68	674.50
	0.5	5226.10	490.72	1996.52	493.30	5235.99	503.91	2016.31	516.53
	0.7	3693.37	322.88	1158.69	324.77	3703.26	336.07	1178.48	347.56

**Table 17:** AIC and BIC of regression models fitted on data generated with ZINB regression model, *n* = 600

		AIC				BIC			
Dispersion	Proportion of Zeros	Poisson	NB	ZIP	ZINB	Poisson	NB	ZIP	ZINB
0.01	0.1	4012.44	3687.82	3762.22	3645.61	4025.63	3705.40	3788.60	3307.96
	0.3	5291.04	3640.69	2944.29	3009.53	5304.23	3658.28	2970.67	3330.99
	0.5	5858.06	3013.56	2408.92	2441.63	5871.25	3031.15	2435.30	2565.70
	0.7	5404.04	2082.93	1681.98	2019.25	5417.23	2100.52	1708.36	1712.98
1	0.1	8325.06	3907.52	7023.07	3898.82	8338.25	3925.11	7049.45	3942.95
	0.3	8638.02	3424.71	5545.94	3388.55	8651.21	3442.29	5572.33	3458.18
	0.5	8249.33	2739.12	4230.17	2700.33	8262.52	2756.71	4256.56	2744.53
	0.7	6855.00	1865.19	2742.46	1851.60	6868.19	1882.78	2768.84	1851.36
5	0.1	17,280.45	3039.27	10,264.64	3042.69	17,293.64	3056.86	10,291.02	3074.23
	0.3	15,597.37	2517.53	8119.98	2520.66	15,610.56	2535.11	8146.36	2550.90
	0.5	13,165.60	1934.56	5896.48	1936.43	13,178.79	1952.15	5922.86	1966.15
	0.7	9776.28	1278.72	3616.01	1280.44	9789.47	1296.31	3642.39	1309.09
10	0.1	22,968.65	2366.89	11,539.50	2370.34	22,981.84	2384.48	11,565.88	2401.25
	0.3	19,981.48	1934.71	9021.47	1937.67	19,994.67	1952.29	9047.85	1968.43
	0.5	16,344.06	1466.35	6497.22	1473.33	16,357.25	1483.94	6523.60	1499.15
	0.7	11,566.87	955.61	3866.63	962.28	11,580.06	973.20	3893.01	987.91

**Table 18:** AIC and BIC of regression models fitted on data generated with ZINB regression model, *n* = 1000

		AIC				BIC			
Dispersion	Proportion of Zeros	Poisson	NB	ZIP	ZINB	Poisson	NB	ZIP	ZINB
0.01	0.1	6684.63	6143.38	5565.15	6052.92	6699.35	6163.02	5594.60	5420.31
	0.3	8824.90	6064.34	4915.65	4924.89	8839.63	6083.97	4945.10	5931.34
	0.5	9777.68	5020.98	4015.15	4043.07	9792.40	5040.61	4044.60	4073.87
	0.7	9023.63	3466.21	2799.39	3385.59	9038.36	3485.85	2828.84	2833.13
1	0.1	13,858.97	6504.03	11,178.92	6486.14	13,873.70	6523.66	11,208.37	6544.02
	0.3	14,382.64	5698.27	9230.42	5630.25	14,397.36	5717.90	9259.87	5724.47
	0.5	13,733.09	4553.36	7032.62	4490.56	13,747.81	4572.99	7062.07	4518.57
	0.7	11,386.64	3105.34	4560.08	3089.41	11,401.36	3124.97	4589.52	3062.81
5	0.1	28,903.48	5055.91	17,208.02	5059.30	28,918.20	5075.55	17,237.47	5094.76
	0.3	26,089.74	4186.58	13,612.61	4189.29	26,104.46	4206.21	13,642.06	4223.62
	0.5	21,943.54	3211.80	9862.55	3213.03	21,958.27	3231.43	9891.99	3244.95
	0.7	16,243.74	2112.63	6053.74	2114.34	16,258.46	2132.26	6083.19	2145.26
10	0.1	38,248.13	3928.96	19,291.56	3932.52	38,262.85	3948.59	19,321.01	3966.42
	0.3	33,318.61	3211.97	15,134.62	3215.36	33,333.33	3231.60	15,164.07	3248.79
	0.5	26,982.05	2431.72	10,754.37	2434.39	26,996.77	2451.35	10,783.82	2467.68
	0.7	19,259.10	1582.60	6502.62	1592.23	19,273.82	1602.23	6532.07	1618.24

The MAEs values of the predicted counts based on Poisson, NB, ZIP, and ZINB regression models fitted on data generated with the ZINB regression model and with different magnitudes of dispersion, zero proportions, and sample sizes are displayed in Tables 19, 20, 21, 22. In scenarios with a small sample size of 50 and dispersion levels of 0.01 and 1, the ZIP model provided the smallest MAEs in nearly all simulation scenarios of proportion of structural zero, except for the scenarios with structural zero proportion of 70% and dispersion level of 0.01. Another exception were the scenarios with structural zero proportion of 10% and dispersion level of 1, where ZINB and Poisson regression models had the smallest MAEs, respectively. In the rest of the simulation scenarios within the small sample size of 50, the Poisson model provided the lowest MAEs (Table 19). As the sample size increased from 200 to 1000, the Poisson regression model no longer resulted with the smallest MAEs. In simulation scenarios with sample sizes 200, 600, and 1000, and a small dispersion of 0.01, the NB produced the lowest MAEs in nearly every scenario of proportion of structural zeros, with the exception when the proportion of structural zeros was 10% where the ZINB in (*n* = 200), ZIP in (*n* = 600) or either NB or ZIP in (*n* = 1000) regression models resulted with the lowest MAEs. Another finding was that in simulation scenarios with large sample size (*n* = 1000), and dispersion level of 1, the NB model produced the lowest MAEs regardless of the proportion of structural zeros. In simulation scenarios with large sample size (*n* = 1000) and dispersion level greater than 1, ZIP model produced the lowest MAEs when the proportion of structural zeros did not exceed 30%; and ZINB produced the lowest MAEs when the proportion of structural zeros was greater than 30%.

**Table 19:** MAE values of regression models fitted on data generated with ZINB regression model, *n* = 50

Dispersion	Proportion of Zeros	Poisson	NB	ZIP	ZINB
0.01	0.1	1.2283	1.2316	1.2274	1.2307
	0.3	3.1310	3.1385	3.1209	3.1402
	0.5	5.2114	5.2070	5.2052	5.2102
	0.7	7.2429	7.2286	7.2366	7.2279
1	0.1	2.2478	2.2833	2.2567	2.2882
	0.3	3.4686	3.5042	3.4598	3.5167
	0.5	5.2457	5.2841	5.2352	5.3016
	0.7	7.2643	7.3470	7.2495	7.3034
5	0.1	4.4559	4.8665	4.5330	4.9490
	0.3	5.0453	5.8211	5.1587	5.9835
	0.5	6.2162	7.2657	6.3823	7.1108
	0.7	7.6836	19.0239	20.2636	22.7636
10	0.1	6.0659	8.2687	6.6112	9.1112
	0.3	6.4278	11.9930	7.0274	9.5274
	0.5	7.2752	14.5155	7.0274	9.5274
	0.7	8.3214	15.8751	14.0274	16.5274

**Table 20:** MAE values of regression models fitted on data generated with ZINB regression model, *n* = 200

Dispersion	Proportion of Zeros	Poisson	NB	ZIP	ZINB
0.01	0.1	1.0586	1.0591	1.0585	1.0565
	0.3	3.1008	3.1002	3.1007	3.1018
	0.5	5.1761	5.1744	5.1759	5.1707
	0.7	7.2169	7.2130	7.2165	7.2146
1	0.1	1.3805	1.3837	1.3802	1.3876
	0.3	3.1443	3.1463	3.1426	3.1504
	0.5	5.1854	5.1823	5.1841	5.1840
	0.7	7.2180	7.2091	7.2166	7.2132
5	0.1	2.3519	2.3926	2.3592	2.4141
	0.3	3.4632	3.5050	3.4640	3.5303
	0.5	5.2182	5.2268	5.2143	5.2361
	0.7	7.2641	7.2626	7.2581	7.2598
10	0.1	3.1613	3.2489	3.1768	3.3383
	0.3	3.9775	4.0793	3.9982	4.1287
	0.5	5.4267	5.5666	5.4403	5.5871
	0.7	7.3654	7.5561	7.3998	7.7825

**Table 21:** MAE values of regression models fitted on data generated with ZINB regression model, *n* = 600

Dispersion	Proportion of Zeros	Poisson	NB	ZIP	ZINB
0.01	0.1	1.0242	1.0242	1.0239	1.0243
	0.3	3.0994	3.0993	3.0994	3.1010
	0.5	5.1722	5.1722	5.1723	5.1716
	0.7	7.2237	7.2233	7.2236	7.2253
1	0.1	1.1257	1.1240	1.1248	1.1245
	0.3	3.1168	3.1164	3.1165	3.1168
	0.5	5.1761	5.1760	5.1761	5.1772
	0.7	7.2476	7.2467	7.2474	7.2470
5	0.1	1.5891	1.5964	1.5911	1.6048
	0.3	3.1700	3.1734	3.1694	3.1776
	0.5	5.2008	5.1966	5.2002	5.1975
	0.7	7.2388	7.2288	7.2372	7.2304
10	0.1	1.9827	2.0020	1.9896	2.0355
	0.3	3.2512	3.2606	3.2539	3.2740
	0.5	5.1940	5.1913	5.1934	5.1893
	0.7	7.2050	7.1947	7.2016	7.1881

**Table 22:** MAE values of regression models fitted on data generated with ZINB regression model, *n* = 1000

Dispersion	Proportion of Zeros	Poisson	NB	ZIP	ZINB
0.01	0.1	1.0317	1.0316	1.0316	1.0317
	0.3	3.1048	3.1048	3.1049	3.1056
	0.5	5.1641	5.1639	5.1641	5.1643
	0.7	7.2360	7.2357	7.2359	7.2351
1	0.1	1.0590	1.0588	1.0583	1.0590
	0.3	3.0980	3.0979	3.0980	3.0990
	0.5	5.1694	5.1688	5.1692	5.1694
	0.7	7.2343	7.2338	7.2343	7.2340
5	0.1	1.2959	1.2958	1.2943	1.3004
	0.3	3.1097	3.1079	3.1089	3.1095
	0.5	5.1660	5.1646	5.1658	5.1644
	0.7	7.2351	7.2316	7.2341	7.2313
10	0.1	1.6692	1.6708	1.6680	1.6957
	0.3	3.1550	3.1544	3.1542	3.1567
	0.5	5.1485	5.1454	5.1479	5.1441
	0.7	7.2436	7.2350	7.2432	7.2339

## Empirical study

### Description of the study population

**Fig. 1:**
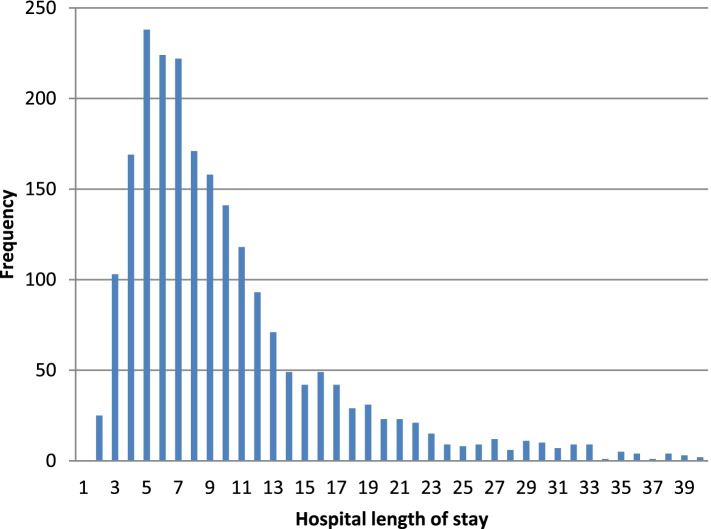
Histogram of hospital length of stay for patients with asthma diagnosis, *n* = 2,167

The empirical study population consisted of 2,167 patients admitted in hospitals with a diagnosis of Asthma selected from MIMIC dataset using ICD-9 code 49,390. Table 23 presents the main demographic characteristics of the study population. Sixty percent of the admitted patients were females. The mean age was 62.3 (SD = 40.66). It should be noted that in the MIMIC data, patients of under89 years old were merged into the same age group. 80.66% of the study population were admitted due to emergency, 17.44% were electively admitted, and just 1.89% of the patients had an urgent type of admission. Most patients had either Medicare (44.35%) or private health insurance (36.41%) (Table 23). The distribution of the variable hospital LOS was positively skewed, with values ranging from 0 to 40 days (Fig. 1). The mean LOS, 8.0 days, was much lower than the variance of 43.10. The larger sample variance compared to the sample mean suggested a deviation from the Poisson regression model’s assumption for equal variance and mean [61].

**Table 23:** Demographic and clinical characteristics of the study population with asthma diagnosis, *n* = 2,167

Characteristic	
Age (years), mean (SD)	62.3 (40.66)
Sex, n (%)	
Male	864 (39.87)
Female	1303 (60.13)
Hospital admission type, n (%)	
Elective	378 (17.44)
Emergency	1748 (80.66)
Urgent	41 (1.89)
Health insurance type, n (%)	
Government	96 (4.43)
Medicaid	304 (14.03)
Medicare	961 (44.35)
Private	789 (36.41)
Self-Pay	17 (0.78)
LOS, mean (SD)	8.0 (6.56)

### Comparison of fitted poisson, NB, ZIP, and ZINB regression models

Table 24 presents the results of fitted Poisson, NB, ZIP, and ZINB regression models for the outcome variable LOS on the patient level predictor variables age, sex, type of hospital admission, and health insurance status. In the zero-inflated models the same predictors were used to fit both the count model and the logistic (zero) model. Based on the results in Table 24, the NB regression model provided the best fit to the data since it resulted the smallest AIC and BIC values. The second-best model was ZINB, followed by the ZIP model. The Poisson regression model resulted with the worst fit to the data according to the AIC and BIC values. The Pearson dispersion statistic in Poisson regression model was 5.3016, greater than 1, suggesting overdispersion. The fitted NB regression model had the smallest dispersion statistic of 1.1815. The regression coefficient estimates and their respective standard errors differed across the models (Table 24). It is quite noticeable in Table 24 the tendency for the Poisson, and ZIP regression models to produced smaller standard errors of the regression coefficient estimates than NB and ZINB regression models. Overdispersion may cause standard errors of the regression coefficient estimates to be underestimated and therefore contributing to discrepancies in significant regression coefficients findings between the models [39, 43]. For instance, at a 5% significance level, only based on the fitted Poisson and ZIP regression models there were significant association between age and log LOS, controlling for the effect of sex, health insurance type, and admission type variables included in the models (Table 24). In relation to the logistic part (zero-model), none of the variables in both ZIP and ZINB regression models had significant contribution to the structural zero-generating process of LOS.

**Table 24:** Findings from fitted multivariable Poisson, NB, ZIP, and ZINB regression models for hospital LOS, *n* = 2,167

Parameter	Poisson		NB		ZIP		ZINB	
	Estimate (SE)	*P*-Value	Estimate (SE)	*P*-Value	Estimate (SE)	*P*-Value	Estimate (SE)	*P*-Value
Age	-0.001 (0.0002)	< .0001	-0.001 (0.0004)	0.0546	-0.001 (0.0002)	0.0002	-0.001 (0.0004)	0.0561
Sex								
Female	0.03 (0.02)	0.1086	0.03 (0.03)	0.4651	0.02 (0.02)	0.1247	0.03 (0.03)	0.4414
Male	reference		reference		reference		reference	
Health insurance								
Government	0.32 (0.11)	0.0045	0.32 (0.21)	0.1228	0.26 (0.11)	0.0215	0.26 (0.21)	0.2129
Medicaid	0.49 (0.11)	< .0001	0.49 (0.20)	0.0131	0.44 (0.11)	< .0001	0.43 (0.20)	0.0319
Medicare	0.46 (0.11)	< .0001	0.46 (0.19)	0.0189	0.41 (0.11)	0.0001	0.40 (0.20)	0.0445
Private	0.41 (0.11)	0.0001	0.40 (0.19)	0.0372	0.35 (0.11)	0.0009	0.35 (0.20)	0.0796
Self-pay	reference		reference		reference		reference	
Admission type								
Elective	-0.30 (0.05)	< .0001	-0.29 (0.12)	0.0159	-0.29 (0.05)	< .0001	-0.29 (0.12)	0.0160
Emergency	-0.17 (0.05)	0.0011	-0.16 (0.12)	0.1649	-0.16 (0.05)	0.0025	-0.16 (0.12)	0.1667
Urgent	reference		reference		reference		reference	
Zero Model	Estimate (SE)	*P*-Value	Estimate (SE)	*P*-Value	Estimate (SE)	*P*-Value	Estimate (SE)	*P*-Value
Age					0.003 (0.004)	0.5015	0.07 (0.13)	0.5722
Sex								
Female					-0.09 (0.43)	0.8437	18.10 (4082.56)	0.9965
Male					reference		reference	
Health insurance								
Government					-14.35 (528.33)	0.9787	-22.23 (16,904.72)	0.9990
Medicaid					-1.55 (1.23)	0.2057	-34.36 (4533.48)	0.9940
Medicare					-1.56 (1.17)	0.1800	-37.75 (4039.53)	0.9925
Private					-2.22 (1.19)	0.0662	-22.36 (5537.88)	0.9968
Self-pay					reference		reference	
Admission type								
Elective					12.99 (810.38)	0.9871	1.81 (20,295.23)	0.9999
Emergency					12.72 (810.38)	0.9873	17.23 (19,158.48)	0.9993
Urgent					reference		reference	
Pearson Chi-Square (value/degrees of freedom)	5.3016		1.1815		4.9001		1.1868	
AIC	17,675.4989		13,134.7955		17,560.4274		13,150.2305	
BIC	17,726.6288		13,191.6065		17,662.6872		13,258.1713	

## Discussion

Simulation and empirical studies were conducted to compare performance in terms of convergence rate and model fit of the Poisson, NB, ZIP, and ZINB regression models. This research added to previous studies by including additional experimental scenarios, such as varying sample sizes, larger dispersion levels, various proportions of zero in the outcome variable, and data generated using Poisson and ZIP distributions, along with NB and ZINB distributions. Our motivating real-life example was the analysis of the count outcome variable hospital length of stay.

Based on the simulation study, when the data were generated with a Poisson regression model (i.e., there was no overdispersion or zero-inflation present in the data), the results showed that regardless of the sample size of the simulated data, the Poisson and ZIP regression models did not have convergence problems. Both NB and ZINB model did not converge 100% in all the sample size simulation scenarios. Compared to NB, ZIP, and ZINB regression models, the Poisson regression model (true model) resulted with the smallest AIC, BIC, and MAE. Our findings slightly differ from the findings reported by Nekesa et al. (2019) where in simulated data with fixed sample size of 500 with no zero-inflation and very low levels of overdispersion, the fitted NB model had the lowest AIC. However, in Nekesa’s study the response variable was generated with a negative binomial distribution [62]. By allowing the variance in the data to be greater than the mean, we generated overdispersed data with a NB regression model varying the level of dispersion and the sample size. We found that when the data have very low overdispersion, the Poisson regression model provided the smallest AIC and BIC statistics values regardless the sample size and fitting a NB or ZINB model may encounter convergence problems especially in situations where the data have low overdispersion. This was expected since as the dispersion parameter approaches zero, the NB distribution approaches the Poisson distribution [63]. When the dispersion was greater than 1, the NB model (true model) provided the best fit in terms of AIC and BIC, regardless of the sample size. These results are in line with Gardner [31] and Saffari [64], who showed that NB regression should be used when there is overdispersion in the data. In the scenarios with large sample sizes (200, 600 or 1000) the NB model produced the smallest MAE regardless of the dispersion level present in the data. Interestingly, when the sample size was 50, the Poisson regression model produced the smallest MAE. The reason for this could be that the small sample size affected the accuracy of the estimation [65]. When manipulating the sample size and the proportion of structural zeros in the data generated with a ZIP regression model, we found that ZINB had less than 100% convergence rate regardless of the sample size or proportion of structural zeros in the data. However, the NB model convergence rate was better than the ZINB regression model convergence rate since NB reached 100% in most of the simulation scenarios. It is important to note that the ZINB regression model is more complex than NB, which may influence the performance of the Quasi-Newton algorithm used for MLE estimations [66]. Other researchers have reported similar convergence issues in fitting the ZINB regression model. Lee et al. (2012) reported that the model did not always converge, or a model diagnostic indicated that the estimated model was not reliable. Another finding was that in scenarios with proportion of zeros of 30% or greater, the ZIP regression model (true model) had the best fit since it had the smallest AIC and BIC statistic values regardless of the sample size. This is in line with the findings reported by Nekesa et al. (2019), where in simulated conditions of very small overdispersion and proportion of zeros equal or greater than 20% ZIP model had smaller AIC than Poisson, NB and ZINB regression models [62]. An interesting finding of our study was that in the simulation scenario with 10% proportion of structural zeros, ZINB regression model had the best fit both in terms of AIC and BIC and regardless of the sample size. With respect to MAE, the performance of the models was very similar. In addition, our findings indicated that in scenarios with the smallest sample size the ZIP regression model had smallest MAE in most of the proportion of zeros scenarios (10%, 30%, 50%). However, when the sample size increased to 1000, the NB had the smallest MAE in most scenarios of proportion of zeros (30%, 50%, 70%). When varying the sample sizes, proportion of structural zeros and level of dispersion in the data generated with ZINB regression model, the ZINB model had convergence issues in the scenarios with small sample sizes. We found that at very low level of dispersion, 0.01, the model that produced the best fit both in terms of AIC and BIC was the ZIP regression model, regardless of the sample size, or proportion of structural zeros. This is not surprising, since, just as the NB distribution converges to the Poisson distribution as the dispersion parameter approaches zero, the ZINB distribution converges to the ZIP distribution as the dispersion parameter approaches zero [63]. In simulation scenarios with sample size greater than 50 and dispersion level fixed at 1, AIC suggested that the best model was ZINB (true model) regardless the proportion of structural zeros in the data. Research based on simulation studies on the use of AIC and BIC for model selection reported that BIC performed better in model selection in the case of large heterogeneity in data due to stronger penalty afforded [67]. In our study, according to the smallest BIC, the NB regression model fit the data better than ZINB in many of the scenarios, depending on the proportion of structural zeros and levels of overdispersion in the data. For instance, when the proportion of structural zeros exceeded 50% and the sample size was greater than 50, and with larger levels of overdispersion both BIC and AIC suggested that NB regression fits the data better. Similar findings were observed in a recent study conducted by Tlhaloganyang et al. [45] that showed that zero inflated models are not always necessary even if the data are characterized by both overdispersion and zero-inflation. Tlhaloganyang et al. [45] reported that the NB model provided a reasonable fit in all datasets when compared to ZIP and ZINB models in over-dispersed and zero inflated data. Similarly, Nekesa et al. (2019) reported results from simulation study and real data analysis of exposed infant diagnosis, showing the negative binomial emerging as the best performing model when fitting data with both structured and non-structured zeros under various settings. Tüzen et al. (2018) reported simulation scenarios, where the NB model outperformed other count models in the presence of outliers and/or excess zeros. Allison (2012) noted that some applications exist in which a compelling case could be made for a zero-inflated model and suggested the use of ZINB instead of ZIP when modeling zero-inflated count data. He stated that the zero-inflated negative binomial model may sometimes fit better than the conventional negative binomial, but for many applications it does not [68]. He recommended that in these cases, it’s important to test for the significance of the difference [68]. Lastly, Hilbe (2014) suggested that the model having substantially lower information test statistic should be preferred, other considerations being equal. In the situations when there is just a slight difference between which models fit statistics, the decision of which model to select should be based on context and how the models are to be interpreted [39].

The results from the empirical data analysis agreed with the findings based on the simulation study. The empirical data were not zero-inflated, and the data had overdispersion based on the Pearson dispersion statistic. The fitted NB regression model had the smallest AIC and BIC values followed by the ZINB regression model. This is in line with the findings of our simulation study, where NB was found to be the best model when dealing with overdispersed data. The Poisson and ZIP models underestimated the standard errors and overstated the significance of some covariates.

Since this study focused on regression analysis methods for count data, in this paper we do not fully discus the findings from the analysis of MIMIC-III data. However, the results from the NB regression analysis of the empirical data demonstrated, that health insurance type and admission type were significantly associated with the log transformed hospital LOS. Patients with elective admission had lower expected number of days of hospital stay compared to patients with urgent admission; and patients with Medicaid, Medicare, or private health insurance had longer expected number of days of hospital stay compared to self-pay patients, controlling for the effect of age and sex. Based on NB regression analysis, Soyiri et al. [69] reported significant associations of sex, age, admission type, ethnicity, week day of admission with asthma LOS in hospitals in London. Based on survey linear regression analysis of asthma Nationwide Inpatient Sample (NIS), collected between 2001 and 2010, Arora et al. [70] reported that white race and private insurance were significantly associated with longer and shorter LOS, respectively. However, the results across these studies are not fully comparable due to difference in variables’ definitions, covariates included in the regression models, type of regression methods, or potential difference in hospitals managements.

Different optimization procedures can produce different results and different rates of convergence. There is no perfect optimization procedure that finds the best solution within the most reasonable amount of time for all sets of data (SAS Institute, 2000). For the SAS programming language, the count regression models would typically be analyzed using PROC COUNTREG, PROC GENMOD, or PROC NLMIXED. The default optimization procedure here is usually the Quasi-Newton or Newton–Raphson. In this study, we used Quasi-Newton method that use iterative approximation and does not require computation of second order derivatives. Hence, it has the advantage of finding solutions quickly. However, this method does not consider the boundary constraints present in the zero-inflated data. Other optimization methods that are often used to fit zero inflated data (as in the case of R software) are the Nelder-Mead Simplex Optimization for ZIP regression models. The use of different optimization methods in the regression models across different software packages may explain some of the differences across studies conducted to evaluate regression methods.

AIC and BIC were used to determine the best model fitted to the data. When comparing the models, it is important to note that the best model will not be necessarily the one with the best fit. Rather, it will be the one that leads to correct inferences, interpretations, and decisions. Although one may not always know the exact model specification that will result in enhanced statistical conclusion, it is still possible to maintain a core principle that the ideal model should be simple and parsimonious [71].

Models’ performance measures were assessed based on the entire sample, but not based on models’ internal and/or external validation. The goal of our study was not to derive predictive modeling function for hospital LOS. The purpose of our study was to illustrate the choice of the count data regression model based on varying combinations of magnitudes of overdispersion, proportions of zeros, and sample sizes*.* In this research, we did not explore other count data regression models, such as Poisson and zero inflated Poisson inverse Gaussian, two-part Hurdle models, zero truncated model, mixture of a binomial and discretized gamma/beta distributions analysis, and others. Hurdle model is a modified count model in which the two processes generating the zeros and the positives are not constrained to be the same. The basic idea is that a binomial probability governs the binary outcome of whether a count variate has a zero or a positive realization. If the realization is positive, the “hurdle is crossed,” and the conditional distribution of the positives is governed by a truncated-at-zero count data model [38]. For example, the Hurdle model may be appropriate to analyze hospital length of stay if the data consisted of patients who were not hospitalized (i.e., zero days of hospital stay) and patients who were hospitalized. In this case, the probability of being hospitalized will be predicted by the logistic regression model and expected LOS will be predicted by the zero-truncated Poisson or NB regression models. Hospital LOS data are rarely zero-inflated. However, studies have reported rising in zero days hospital admissions (i.e., hospital stays of less than 24 h) in pediatric patients and in admissions with URTI/viral infection, gastroenteritis, croup, bronchiolitis, asthma, tonsillitis, non-specific abdominal pain, constipation, febrile convulsion, and rash diagnoses [69, 72, 73]. This may reflect a combination of factors including availability of more rapid assessment and effective treatment of acute presentation and declining hospital expertise and resources [72]. Further research should be conducted for scenarios of different data generating mechanisms in inpatient hospital LOS. Also, in this research we did not explore underdispersion. Even though is not as common to find underdispersed data in real life datasets, it would be interesting to evaluate the performance of count regression models in modeling such distributions. LOS can be analyzed as right-censored time-to-event data using survival analysis methods, where the event of interest is time to hospital discharge, or time to clinical stability, or time to death [74, 75]. If the interest is in estimating the probability of a patient reaching clinical stability or hospital discharge by a given day, Brock, et al. (2011) argued about right-censoring or disregarding the data for individuals who die prior the events. An alternative approach to analyze LOS time-to-event data with multiple events is to treat the events as a competing risk [75, 76]. Competing risk analyses extend survival analysis methods to situations with multiple possible events, where the occurrence of one either precludes the others or substantially alters the probability of other events [75, 76]. If additional measures such as, vital signs to monitor patients during hospitalizations or possible destinations after the first day of admission are obtained, Markov models can be utilized to capture the temporal sequences of events [77–79]. In this study we did not evaluate the extensions of the class of GLMs for analysis of count correlated data collected from multiple observations on individuals or count data that are clustered due to clustered survey data, family studies, or nested experimental designs. The generalized estimating equations (GEEs) [80] and the generalized linear mixed models (GLMMs) [81], also known as random effects models, multilevel, or hierarchical models, are used to deal with such clustered data and produce accurate regression coefficients and standard errors estimates. The technique of multilevel modeling incorporates cluster specific random effects to accounts for this within cluster dependency by partitioning the total data variance into between and within cluster variation [82]. For example, multilevel modeling will be appropriate if LOS data are collected from various participating hospitals with available hospital level variables and there is a systematic between-hospital variation in patient outcome; or LOS data are collected for the same subjects at multiple time points and there is a systematic between-subject variation in patient outcome. Whether a multilevel model is needed can be inferred from either the intraclass correlation coefficient (ICC) and/or the significance of the random effect variance component for the clustering variable in the null model. GEE methods account for correlation by incorporating predefined “working” correlation structures to describe the nature of within-clusters dependencies [80].

## Concussions

The Poisson and ZIP regression models performed poorly in over-dispersed data. ZIP outperformed the Poisson, NB, and ZINB regression models when there is just zero-inflation but no overdispersion in the data. NB model provided the best fit in over-dispersed data and outperformed ZINB model in many cases of both zero-inflation and overdispersion. Just a slight difference existed between the fit statistics of NB and the more complex to fit and interpret ZINB model. The researcher should decide if a zero-inflated regression model is more appropriate to model the data. If the researcher believes there are two different data generating mechanism producing zeros, then the NB regression model may not capture the different characteristics of the two groups generating the zeros and, in this case, the ZINB regression model could provide greater flexibility when modeling the zeros. In addition, NB and ZINB regression models faced substantial convergence issues when incorrectly used to model equidispersed data. It is important to check for ovedispersion. Fitting incorrect models to overdispersed data leaded to incorrect regression coefficients estimates and overstated significance of some of the predictors.

Although the work presented here is based on the analysis of hospital LOS, the findings from the simulation study are generalizable to other count outcome variables. Our findings can guide in the selection from the studied generalized linear models in the development of hospital and public health analytical applications for the computation of risk-adjusted LOS.

## Data Availability

The datasets used and/or analyzed during the current study available from the corresponding author on reasonable request.

## Abbreviations

AIC: Akaike’s information criteria
BIC: Bayesian information criteria
COVID-19: Corona virus disease 2019
GEE: Generalized estimating equation
GLM: Generalized linear model
GLMM: Generalized linear mixed model
ICC: Intraclass correlation coefficient
ICD-9: International classification of diseases, ninth revision
LOS: Length of stay
MAE: Mean absolute error
MIMIC: Medical Information mart for Intensive care
MLE: Maximum likelihood estimation
NB: Negative binomial
NIS: Nationwide inpatient sample
SAS: Statistical analysis system
ZINB: Zero-inflated negative binomial
ZIP: Zero-inflated Poisson
